# Reinforced Smart Foams Produced with Time-Profiled Magnetic Fields

**DOI:** 10.3390/polym13010024

**Published:** 2020-12-23

**Authors:** Daniele Davino, Marco D’Auria, Roberto Pantani, Luigi Sorrentino

**Affiliations:** 1 Dipartimento di Ingegneria, Università degli Studi del Sannio, Piazza Roma 21-24, 82100 Benevento, Italy; davino@unisannio.it (D.D.); marco.dauria@unina.it (M.D.); 2 Istituto per i Polimeri, Compositi e Biomateriali, Consiglio Nazionale delle Ricerche, Piazzale Enrico Fermi 1, 80055 Portici (NA), Italy; 3 Solar Thermal and Smart Network Division, ENEA—Italian National Agency for New Technologies, Energy and Sustainable Economic Development, Via Anguillarese 301, 00123 Rome, Italy; 4 Dipartimento di Ingegneria Industriale, Università di Salerno, Via Giovanni Paolo II 132, 84084 Fisciano (SA), Italy; rpantani@unisa.it

**Keywords:** smart foams, magnetic particles, variable magnetic field, magnetoelasticity

## Abstract

Polymeric smart foams are lightweight and multifunctional porous materials that are sensitive to the magnetic field due to the presence of magnetic particles embedded in the matrix. Recently, a constant magnetic field has been exploited to align the particles along the magnetic field lines during the formation of the porous structure. In this paper, a new field-structuring process was developed that makes use of a time-profiled magnetic field during the foaming process to control the geometrical features of the particles aggregates. The effects of magnetic field strength as well as the switch-on and switch-off times on the magnetoelastic behavior of the smart foams were investigated. It was proven that the alignment of the particles results in both a strong relative sensitivity to the magnetic field and a positive stress change, whose extent depends on the geometrical features of the developed aggregates.

## 1. Introduction

Smart foams are magnetoelastic (ME) materials that are sensitive to an external magnetic field and exhibit a fast and reversible change in the mechanical response [1]. The adjustable mechanical properties of ME materials allow for their use in different applications, such as adaptive variable-stiffness devices, soft actuators, and artificial muscles [2,3,4]. Among ME materials, those based on polymers—such as elastomers, gels, or foams—have the advantage of being easily moldable and scalable in performance. The sensitivity to the magnetic field is generally developed by adding magnetic particles in the polymer precursors during a preliminary mixing step. It is apparent that both mechanical and magnetomechanical responses, as well as the functional features (for instance EMI shielding, thermal, or electric conductivity), are dependent on the peculiar spatial distribution of the particles, which is set during the consolidation process of the polymer in a mold. Then, any effort in the improvement of this process phase may lead to significant results in the functional performances.

The simplest configuration of particles in a magnetosensitive polymer composite is the random distribution, which is easily obtained by well dispersing the particles in a low viscosity polymer before its consolidation. Such materials are characterized by both mechanical and functional isotropy. More advanced systems are produced by field-assembling the magnetic particles along preferential directions (aligned particles). It has been proven that the application of a constant magnetic field during the production process allows the particles along the magnetic field lines to be assembled in order to form chain-like structures [5]. More complex constant magnetic field configurations [6] or mechanical treatments applied to the polymer before the consolidation step [7] have been used to prepare ME materials with a planar spatial distribution of particles.

ME solids based on elastomeric polymers with aligned magnetic particles have been developed since 1999. Such attempts were made by using high viscosity polymeric precursors, which needed very high magnetic fields (up to 1 Tesla) to effectively distribute the particles during the consolidation process [8]. Moreover, ME elastomers still require very high magnetic fields to exploit their magnetoelastic response after consolidation, due to the high elastic modulus and density of the matrix. Typical achievable deformations are of the order of a magnitude of parts per million, as theoretically and experimentally shown [9,10,11].

In order to overcome the limitations of magnetoelastic solids (i.e., material density higher than 3000 kg/m^3^, high elastic moduli, high viscosity matrix during the particles manipulation by the magnetic field), ME polymeric foams were developed. They combine the lightness of porous structures with the lower magnetic field requirements in order to (a) induce the alignment of the particles (low viscosity matrices) [12,13,14,15,16,17] and (b) exploit the peculiar functional properties [18,19,20,21,22], which depend on the polymeric matrix and the degree of porosity of the cellular structure. ME foams open to the possibility of producing lightweight smart structures for applications requiring low forces and large deformations, high sensitivity, and low weight.

ME foams exhibit a magnetomechanical response after the consolidation of the polymer. The application of a magnetic field can result in either a magnetostrictive effect or a change in the apparent elastic modulus [8]. The magnetostrictive effect consists of the change of the shape along the field direction. The magnetostriction can be positive (foam elongation) or negative (foam contraction), and this has been investigated from the experimental [8,9,10,11,23,24], analytical [25,26,27,28], and numerical [29,30,31] perspectives. The effect of the magnetic field on the elastic modulus of a magnetosensitive polymer has been studied, and a direct proportionality of the static compressive or shear moduli with the magnetic field amplitude has been reported [24,32,33,34,35,36,37,38]. Both effects can be related to the induced dipole–dipole interactions among magnetic particles with the polymer. The magnetic field causes attraction or repulsion between particles according to their mutual positions with respect to the magnetic field lines, such as shown in simulations [31] and experiments [33,39]. More recently, the role of particle shape was also investigated [40].

In this work, lightweight smart materials are prepared by field-structuring the magnetic particles by means of a time-profiled magnetic field within the foaming time window. The use of a magnetic field changing in time improves the flexibility of the production process with respect to the constant field methods. It is demonstrated that the performance gain can be tailored to specific applications requirements. A very important capability is the possibility of controlling the local magnetic field sensitivity for both static and dynamic performances in large-strain/low-force applications by simply changing the local degree of anisotropy in the field-structured material during the production process.

## 2. Materials and Methods

### 2.1. Materials

A commercial grade polyurethane (PU) formulation was used as a polymeric matrix for the foams. A polyether polyol (Elastoflex W 5105/172, density equal to 1.03 g/cm^3^, hydroxyl number 55 mgKOH/g), which provided all chemical agents (water, catalysts, and surfactants) for the foaming reactions, and a 4,4’-Methylene diphenyl diisocyanate (ISO 135/111, density equal to 1.20 g/cm^3^, NCO content equal to 29.5%), both supplied by BASF Poliuretani Italia (Villanova d’Asti –AT, Italy), were used at a 100:53 ratio to produce the PU foam. Carbonyl iron particles (CIP; grade SQ-R, mean particle size 5 μm—D50, particle surface treated to avoid particle corrosion and supplied by BASF SE, Ludwigshafen, Germany) were used as magnetic particles. The iron particles are for electronic applications, and they exhibit a relative magnetic permeability of about 20 and a high saturation magnetization (above 1 T).

### 2.2. Foams Preparation

CIP particles (25% by weight, 3.3% by volume with respect to the polymer) were dispersed in the polyol and stirred for 5 min at 2000 RPM by means of a homogenizer to be evenly dispersed. The diisocyanate was then added and intimately mixed for 10 s before pouring the reacting mixture in a 120 mm × 120 mm × 20 mm aluminum mold. The foam was kept in the mold for 10 min, after which the curing process was finalized in an oven at 40 °C for 24 h.

**Figure 1:**
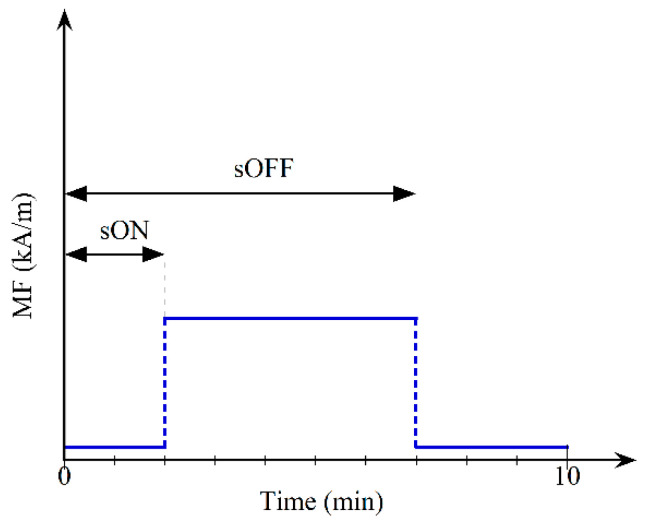
The magnetic field time profile (blue line) is schematized with switch-on (sON) and switch-off (sOFF) time parameters.

The spatial distribution of particles within the matrix was manipulated during the foaming process by means of a variable magnetic field (MF), which aligned the particles along the magnetic field lines to form elongated, chain-like structures. The magnetic field time profile was generated by means of a custom-made C-shaped electromagnetic dipole, built from laminated iron sheets (see Appendix A.2 for more details on the setup), with a 100 mm × 100 mm × 40 mm airgap. The magnetic field intensity was varied in time to impart the desired geometrical features to the field-structured aggregates. In order to show the potential of such approach, the magnetic field time profile was managed by changing the amplitude, the switch-on (sON) time, and the switch-off (sOFF) time, as sketched in Figure 1. The time increment for the sON and sOFF parameters was set as 1 min to guarantee enough significance and repeatability to the experiments. A further value for sON was added (0.5 min) due to the high reaction rate in the first part of the foaming process.

The effect of CIP particles on the reaction kinetics was evaluated by means of the FOAMAT instrument in specific tests performed in an open mold (Format Messtechnik GmbH, Karlsruhe, Germany). The recorded parameters (rise height, H; reaction temperature, T; pressure, P; dielectric polarization, D) were used to compare the foaming process in the presence and in the absence of magnetic particles.

An overview of the investigated samples series is shown in Table 1. The total PU processing time was kept constant at 10 min. The magnetic field strength and the magnetic field time profile were changed to identify the effects of the aggregates’ morphology on the elastic and magnetoelastic responses. In order to better identify the effects on the smart foam performance, the MF parameters were changed one at a time.

Samples were coded by specifying the composition identifier (i.e., PU for neat PU foam, RF for foams with randomly dispersed particles, AF for foams with aligned particles), the magnetic field strength in kA/m that was used during the foaming process, as well as the switch-on (sON) and switch-off (sOFF) times (in minutes; times are measured starting from the mold insertion in the airgap). For instance, AF_189_T0-10 refers to a foam sample with the aligned particles produced under a magnetic field 189 kA/m in strength, applied from minute 0 to minute 10.

### 2.3. Characterizations

The morphology of the foams was investigated by using an optical microscope (PlanApo MZ16, from Leica MicroSystems, Wetzlar, Germany). The density of the samples was calculated as the ratio between the mass and volume, averaged on five samples 50 mm × 20 mm × 20 mm in size. The samples were cut from the center of the foamed slabs to avoid boundary effects related to the inhomogeneity of the cellular structure close to the mold frame.

A three-dimensional microtomographic analysis was performed on three representative samples to identify the cellular structure and the spatial distribution of the particles in field-structured foams at different strain levels. Tomographic images were acquired through the SYRMEP BeamLine at the ELETTRA synchrotron facility (ELETTRA Sincrotrone Trieste SCpA, Trieste, Italy), and 3D volumes were reconstructed with SYRMEP Tomo Project software (ver. 1.4) [41].

Mechanical tests were performed on samples measuring 50 mm × 20 mm × 20 mm in size by using a universal testing machine (model 4304 from SANS—Shenzen, China, now MTS, Eden Prairie, MN, USA) equipped with a 5 kN load cell. The strain rate was set equal to 10^−2^ s^−1^. The compressive response was evaluated in the foaming direction (in AF foams this coincides with the particle alignment direction) according to ASTM 1621-16. Five samples for each foam composition were tested to statistically assess the effect of particles’ content and their distribution on the compressive behaviors.

**Figure 2:**
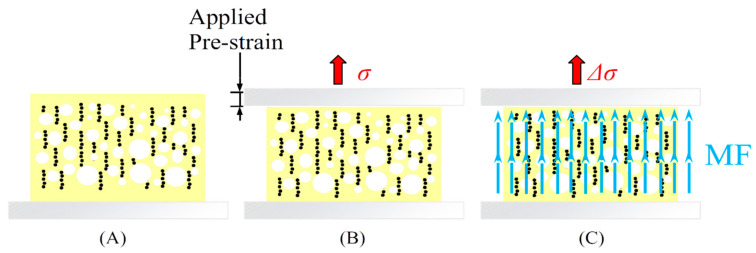
Magnetoelastic test steps: (**A**) sample positioning, (**B**) prestrain application and stress evaluation (*σ*), (**C**) stress change evaluation (Δ*σ*) under the sinusoidal magnetic field (MF).

A further custom-made experimental setup was specifically designed to apply the MF on specimens during the magnetoelastic characterization (see Appendix A.2 for details). Magnetoelastic tests were performed on samples 50 mm × 20 mm × 20 mm in size. A specific procedure was defined to evaluate the magnetoelastic response (Figure 2). First, a prestrain ranging between 2 and 20% was applied along the foam-growth direction. The stress was evaluated along a delay time of 20 min to allow for the stress relaxation of the polymer. After the stress relaxation rate is below 0.01 kPa per minute (Figure 2B), the magnetoelastic response was evaluated by applying a sinusoidal magnetic field (132 kA/m in strength, 0.05 Hz in frequency) at each fixed prestrain value (Figure 2C) [14].

## 3. Results and Discussion

### 3.1. Foam Preparation and Morphology

**Figure 3:**
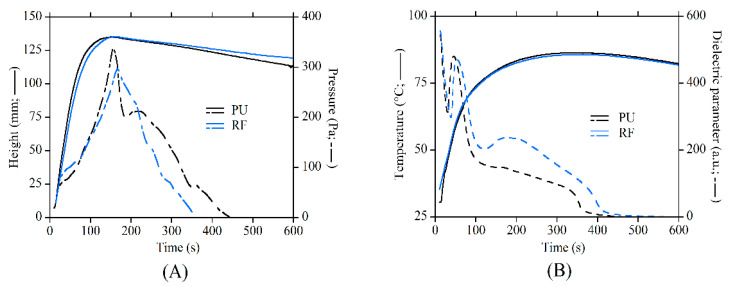
Comparison of the processing parameters from the FOAMAT instrument recorded during the foaming of the PU (neat PU foam) and RF (foams with randomly dispersed particles) systems: (**A**) temperature and pressure; (**B**) height and dielectric polarization.

The relevant parameters of the reaction kinetics in the presence and absence of particles were evaluated in order to check whether magnetic particles could affect the reactivity of the polyurethane and consequently the development of the foam morphology and the mechanical response of the polymeric phase. The PU and RF systems show similar trends in all the relevant parameters. The height (Figure 3A) and temperature (Figure 3B) curves are fairly close. The pressure parameter takes into account the pressure that builds up in the foam after the reacting components have set, and it was measured under the sample by a specific sensor. The dielectric parameter was determined by chain-like molecules having a large dipole moment due to their polar ends (OH, NCO), whose value depends on the number of available dipoles during the curing reaction between the diisocyanate and polyol molecules. Both the pressure and dielectric parameters are slightly different between the PU and RF systems. The dielectric parameter allows us to indirectly estimate the residual reactivity of the polymer, and it decreases to zero with the reduction of free hydroxyl groups. As long as the reaction proceeds, the dielectric parameter shows an excess of available dipoles in the RF with respect to the PU, while the pressure curve of the RF is slightly lower. Both parameters indicate a marginally lower curing rate in the RF, but the shape and the time features of the curves are very similar. As a consequence, the foaming processes in the absence and in the presence of CIP particles were considered analogous.

**Figure 4:**
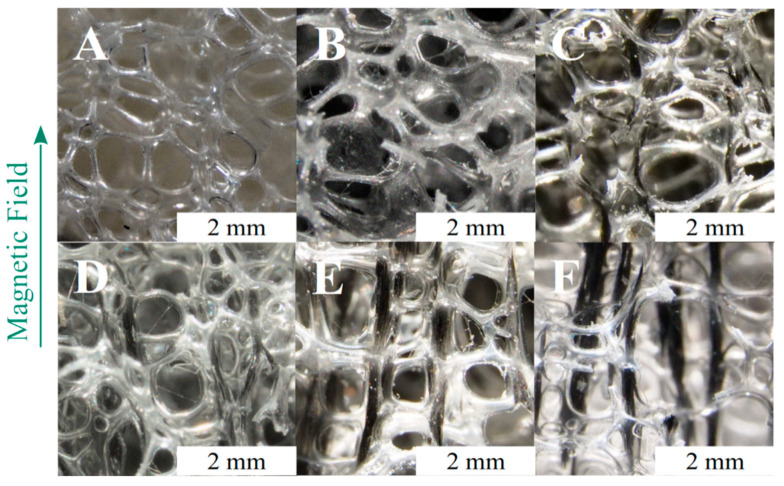
Optical micrographs of the following samples: (**A**) PU, (**B**) RF, (**C**) AF_24_T0-10, (**D**) AF_48_T0-10, (**E**) AF_96_T0-10, (**F**) AF_189_T0-10. The scale bar is the same for all samples.

**Figure 5:**
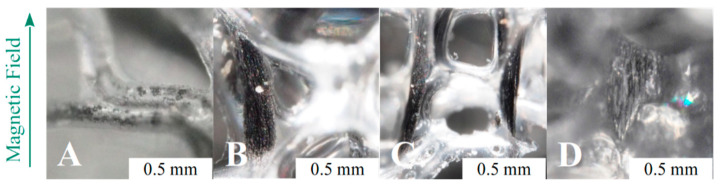
Details of the particles aggregates in samples after different MF time profiles: (**A**) RF, (**B**) AF_48_T0-10, (**C**) AF_96_T0-10, (**D**) AF_189_T2-10.

**Figure 6:**
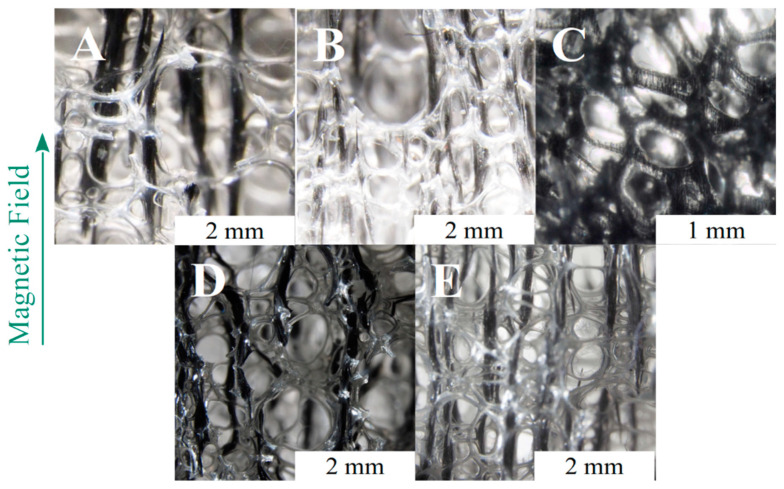
Optical micrographs of the following samples: (**A**) AF_189_T0-10, (**B**) AF_189_T0.5-10, (**C**) AF_189_T2-10, (**D**) AF_189_T0-1, (**E**) AF_189_T0-2.

All samples show an open-cell morphology and a regular porous structure, with filled systems showing the same morphological features of PU (Figure 4A). Although the apparent density of reinforced foams is higher because of the presence of the CIP particles, the porosity is comparable between all systems (relevant samples parameters are reported in Table A1). Therefore, it is confirmed that the proposed production process allows a linear aggregation of CIP particles to be developed in parallel with the magnetic field lines without worsening the regularity of the cellular morphology in terms of porosity, cell shape, and mean cell size (see Figure 4, Figure 5 and Figure 6).

The particles in RF (Figure 4B) were randomly dispersed, and no ordered structures can be identified (Figure 5A). A first series of foams with aligned structures was produced by changing the MF strength during the foaming process. The MF was applied from the start of the foaming process (sON equal to 0 min) up to de-molding (sOFF equal to 10 min). The optical micrographs of the samples show that the magnetic particles were assembled in ordered structures even at the lowest MF strength (24 kA/m, Figure 4C). They are curvilinear and positioned around the cell struts, and their size is similar to the bubble size. The low intensity magnetic field was not able to overcome the distancing action of the expanding polymer, and the final shape of the aggregates conformed to the shape and size of the growing bubbles. As soon as the magnetic field strength was increased to 48 kA/m, the aggregates’ length increased, and the shape became more linear (Figure 4D). At 96 kA/m, the aggregates are almost perfectly linear and are finely distributed in the observed volume. Their size is well above the mean cells size (Figure 4E). Finally, the application of an MF equal to 189 kA/m results in thick structures and coarse spacing with respect to the other field structured foams (Figure 4F). The size of the aggregates is typically 10 times the cells size, and several are as long as the foam sample (20 mm). As a general conclusion, the increase of the MF strength allowed longer and thicker aggregates to be built, but too high MF values can induce coarsening of the field-structured aggregates [42].

The dielectric parameter from the FOAMAT analysis shows that the polyurethane reactivity strongly reduces after 120 s, with the height almost peaking at such time. Although the reaction continues up to 350 and 400 s for the PU and RF systems, respectively, the polymer viscosity is high enough for us to set the final volume of the foam after 2 min. A direct consequence of the viscosity increase is the reduction in the capability of particles to move through the polymers. For this reason, a second set of magnetoelastic foams was produced by delaying the MF switch-on time (sON equal to 0.5, 1, or 2 min) with the aim of understanding to what extent the MF can be delayed so that it can still build linear aggregates. A delay in the switch-on time implies that the foam develops as RF foam from the beginning of the reaction up to sON, and then it develops into a field-structured foam from sON to de-molding. It is evident from Figure 6B that a delay of 30 s (sON = 0.5) results in a reinforced foam with long and finely dispersed aligned aggregates that are higher in number but thinner with respect to sON = 0 (Figure 6A). This can be explained by considering that the viscosity of the polymer has already increased after 30 s, and the difficulty to move the particles for long distances through the polymer increases. The assembling of aggregates is still effective but proceeds along the field lines at a shorter range. This phenomenon is even more marked at sON = 1, when smaller and thinner aggregates are developed. Interestingly, at sON = 2, the linear aggregates are not visible to the naked eye, unlike those in the other aligned cases (Figure 6C). This occurs because the viscosity is too high to allow for any effective movement of particles within the polymeric matrix, and the particles’ aggregates macroscopically resemble random distribution (Figure 6C). This result is in excellent agreement with the FOAMAT curves from the polyurethane reaction kinetics (Figure 3). Nevertheless, ordered structures are present at the microscale within the single foam struts (Figure 5D).

Changes in sOFF allow us to understand whether the particles alignment induced at the beginning of the foaming process (sON = 0) can be kept after the magnetic field removal (Figure 6D–E). The early switching off of the MF (sOFF = 1) allowed aligned aggregates to be obtained after foam setting but they are thick and short. The strong magnetic field allowed the particles to effectively move through the low viscosity polymer but the bubbles growth process broke the aggregates into short and low aspect ratio structures that lay along the cell struts and edges (Figure 6D). A longer application of the magnetic field (sOFF = 2) resulted in longer aggregates with respect to sOFF = 1 (Figure 6E), while the thickness is the same.

### 3.2. Static Mechanical Behaviour in Compression

**Figure 7:**
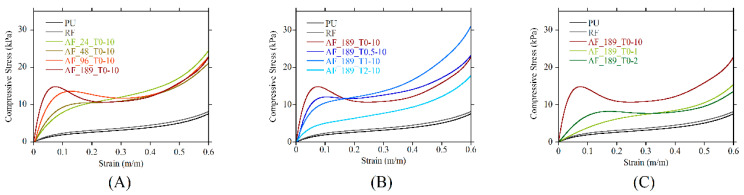
Representative stress–strain curves of foams as function of: (**A**) magnetic field strength, (**B**) sON, (**C**) sOFF.

The mechanical response of the foams is shown in Figure 7A–C, while the main mechanical parameters are reported in Table 2. According to the ASTM 1621-16 standard that was considered for the foam characterization, if no stress peak is present in the stress– strain curve, then a conventional 10% yield strain has to be considered, and the yield stress has to be calculated according to such strain. The direct relationship between the mechanical response and the magnetic field applied during the foaming process is evident. In particular, the increase in the MF intensity improves both the compressive modulus and yield stress (Figure 7A). This behavior can be related to the thickness and length of the aggregates. In the case of randomly dispersed particles, either single particles or few-particle random aggregates are present, and a marginal increase in the elastic response (compressive modulus and yield stress) with respect to the PU is induced due to the low volume content of particles in the RF. AF systems are much better performing than RF ones because the elongated reinforcing aggregates act as fibers, and the performance gain is up to 12-fold in the compression modulus and up to 5-fold in yield stress with respect to the equally composed RF. Foams produced under the highest magnetic field strength (AF_189_T0-10) show a clear relative maximum after the elastic range, followed by a stress drop. This is due to the very long and thick linear aggregates crossing almost the entire sample, which incurs buckling just below 8% of the strain [42].

The delay of the MF application during foaming (sON > 0) changes the morphology of the aggregates (Figure 6), and this reflects the mechanical response (Figure 7B). The stress–strain curve shape radically changes just with a 30 s delay (sON = 0.5). The compressive modulus and the yield stress become lower with respect to the nondelayed system, but the stress peak disappears and no stress drop is detected. A stress plateau up to a 30% strain is evident, in place of the stress drop to a relative minimum present at sON = 0. The stress drop disappearing is due to the increased number of structures that are aligned transversally to the compression direction per unit surface, which more effectively supports the cellular structure and limits the buckling of the reinforcing aggregates [42]. At sON = 1, the compressive modulus and the yield stress further reduce, but the stress rises at a higher rate just after the curve knee at around a 6% strain. At sON = 2, the mechanical behavior is similar in shape to sON = 1, but it is significantly shifted towards lower values. This is due to the small size of the reinforcing structures.

The change in the switch-off time of the MF allowed us to verify whether the field-assembled linear aggregates can keep their features after being formed. It is evident in Figure 7C that the mechanical response at sOFF = 2 is largely reduced with respect to sOFF = 10. Indeed, the analysis of the morphology shows that the aggregates are always formed under the MF, but their size is reduced due to the bubble expanding process, which can break the formed aggregates in thick but shorter reinforcing structures when the magnetic field is removed. At sOFF = 1, the mechanical response is further depleted. The very short length of the aggregates, in this case, does not allow for a large reinforcing effect.

**Figure 8:**
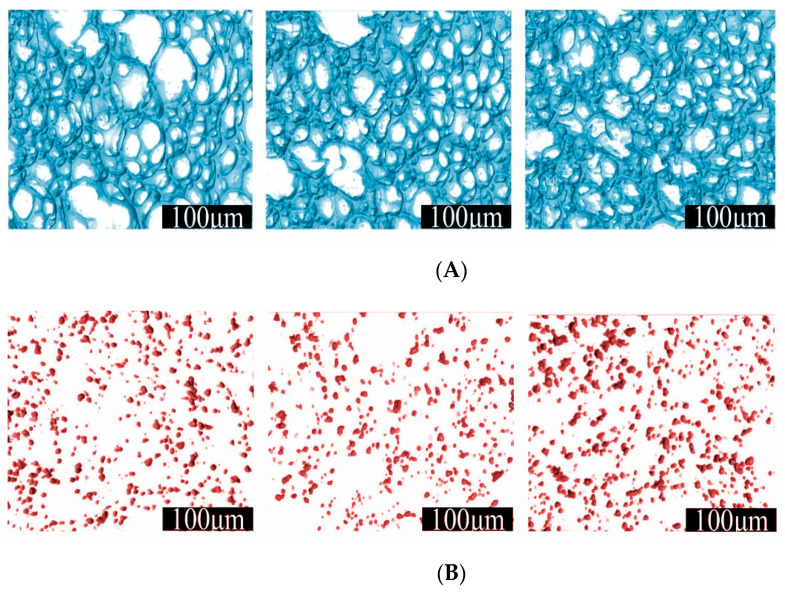

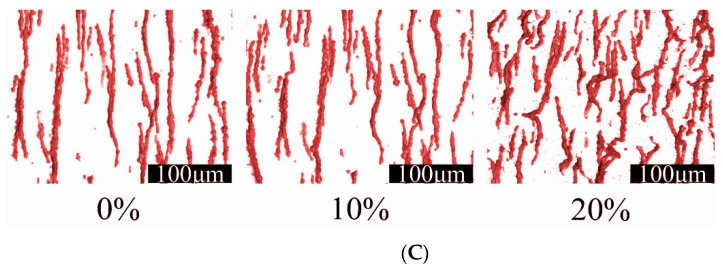
Microtomographic reconstructions at different strain levels of three representative samples: (**A**) PU, (**B**) RF, (**C**) AF_189_T0-10.

A microtomographic analysis was performed on selected samples (Figure 8A–C) at three strain levels (0%, 10%, and 20%) to correlate the mechanical response with the morphological features under compression. The compression of the foam induces a change in the morphology of both the cellular structure and reinforcement. The cellular structure of the neat PU foam is regular and shows the characteristic cell size distribution of foams (Figure 8A). The compression test induces distortion and buckling of the struts. These phenomena are barely detectable at the 10% strain, while they are evident at the 20% strain, at which the cell shape is markedly irregular. The particle distribution in the RF increases in volumetric density during compression. The qualitative distribution of the particles does not change, but the particle mean distance reduces (Figure 8B). Foams with aligned particles show a very different particle distribution (Figure 8C). In fact, linear aggregates can be clearly identified, which are aligned along the magnetic field lines. Compression deforms the aggregates shape, but at the 10% strain, only a reduced buckling is detected. The aggregates shape marginally changes because the aligned structures are still linear and oriented in the same fashion, unlike at a 0% strain. At the 20% strain, the aggregates undergo large buckling. Their shape deviates from the pristine vertical alignment and appears as segmented.

### 3.3. Magnetoelastic Behaviour

The magnetoelastic characterization shows the smart behavior imparted by linear aggregates. This was performed by measuring the stress change under a sinusoidal MF (amplitude equal to 132 kA/m, frequency 0.05 Hz) at different prestrains (2%, 5%, 10%, 20%). Both the strain and the MF were applied along the foaming direction, which is coincident with the alignment direction of the aggregates during the foaming process. The stress induced by the MF was combined with the polymer response, and therefore it can be evaluated by subtracting the stress value just before the application of the MF from the actual stress value measured under the MF.

**Figure 9:**
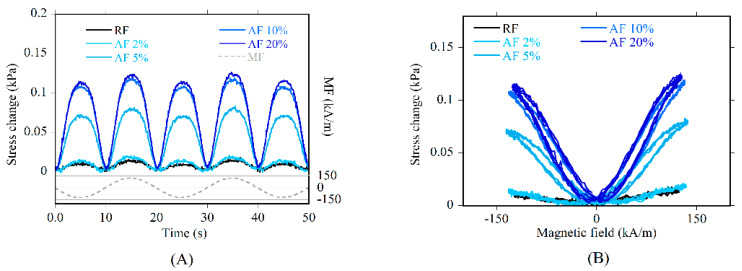
Stress change under magnetic field versus (**A**) time and (**B**) magnetic field for RF (at 2% prestrain) and AF_189_T0-10 at different prestrains.

The PU did not show any stress change in response to the application of MF, as expected. Figure 9A shows the comparison of the stress variation detected in RF and AF_189_T0-10. It is evident that the stress continuously followed the applied magnetic field. It is worth noting that the stress signal has a double frequency with respect to the MF signal (dashed line in Figure 9A). CIP particles are iron-based, and their magnetization follows the MF regardless of the field direction. As a consequence, the stress variation exhibited a peak in occurrence of each minimum and maximum of the MF sinusoidal signal [16].

In order to represent the smart behavior of AF foams, the butterfly chart for the AF_189_T0-10 sample at different prestrains is shown (Figure 9B). AF systems had a significant magnetoelastic response, which depends continuously and proportionally on the MF signal amplitude, in coherence with iron-based magnetostrictive materials. The stress change is independent of the direction of the MF, being always positive. Such an effect is regarded as an apparent change in the foam stiffness (ΔE-effect).

**Figure 10:**
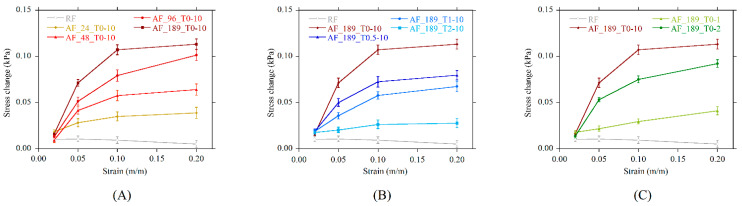
Stress change under magnetic field versus prestrain as a function of (**A**) magnetic field strength, (**B**) switch-on time, (**C**) switch-off time.

RF exhibited a limited sensitivity to the MF, since its stress change was small, and it decreased with the increase of the prestrain. This behavior can be related to the equilibrium between attractive and repulsive forces dependent on the mutual distance between the pseudospherical aggregates within the sample volume [43]. The increase of the prestrain reduced the mutual distance between particles, and this can enhance the attractive forces shared. By contrast, AF foams behaved differently. Their sensitivity to the MF is comparable to that of the RF only at the 2% prestrain, while it strongly increased at higher prestrains (Figure 10A). The highest magnetoelastic response was detected in systems with thicker and longer aggregates, produced under 96 and 189 kA/m. The stress change increased monotonically with the prestrain, even if a plateau can be identified above the 10% prestrain in most of the systems.

Systems produced by delaying the MF switch-on time (B-series samples) are characterized by the same performance trend with a prestrain with respect to those in the A-series. The lower stress change detected in samples produced with sON > 0, and hence the reduced sensitivity, is a consequence of the shorter and thinner aggregates, which had a less pronounced interaction with the magnetic field (Figure 10B).

Samples prepared by reducing sOFF exhibited a decrease in the response to the MF in proportion to the switch-off time (Figure 10C). This is again in very good agreement with the morphology of the aggregates, which were shortened by the breaking action of bubble growth after the removal of the MF. It can be seen that a short MF application time from the beginning of the curing reaction (2 min) allowed for the production of foams with a magnetoelastic performance comparable to the system produced with sON = 0.5 (9.5 min MF application time). Considering that less but thicker aggregates were detected in AF_189_T0-2 with respect to AF_189_T0.5-10, it follows that thicker aggregates have a stronger interaction with the magnetic field.

The increase of the stress variation with a prestrain is due to the fact that linear aggregates deviate from the linear shape and buckle in proportion with the deformation. The application of the magnetic field promotes the realignment of such buckled structures along the MF lines. The tendency to recover the straight shape is the reason of the detected stress change. Above the yield point, this effect was still present but a sort of saturation arises, since the stress change at the 20% prestrain was only slightly higher than that at the 10% prestrain. This phenomenon can be related to the change in the shape of the aggregates during compression, as shown by the 3D microtomographic analysis (Figure 8C). Some of the long linear aggregates were broken into segmented structures, characterized by small linear portions joined together by transversal short linear aggregates (Figure 8C, 20% strain). These structures, if not increasingly deviating from the alignment direction, would result in an increased MF-related stress change. Therefore, the occurring segmentation has the effect of reducing the effective length along the MF lines. The overall result is that the sensitivity to the magnetic field was lower than expected. The saturation can be considered as the combined effect of two stress contributions: (a) the strong contribution (full response) of long and bent aggregates, and (b) the weak contribution of the occurring small aggregates coming from the segmentation of long ones.

**Figure 11:**
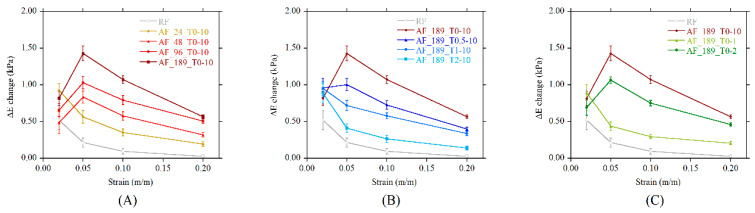
The change in the apparent modulus (ΔE-change) versus the prestrain as a function of (**A**) magnetic field strength, (**B**) switch-on time, (**C**) switch-off time.

In order to understand the effect of the geometrical features of the aggregates, two normalized parameters can be calculated, namely the change in the apparent modulus (ΔE-change) and the relative stress change (RSC). The ΔE-effect is indicative of the capability of the system to show incremental sensitivity to the MF with strain. It was calculated as the ratio between the stress change and the actual prestrain value, and it is plotted for all systems in Figure 11A–C. Systems with long aggregates (obtained with a magnetic field higher than 48 kA/m, sON < 1, sOFF >1) show a marked increase in the ΔE-change in the linear elastic region (2% and 5% prestrain), i.e., an increased sensitivity to the MF (Figure 11A). Thin and short aligned structures, on the contrary, are responsible for the reduction in sensitivity with strain (Figure 11B,C). The ΔE-change decreased in all systems at prestrain values outside the linear elastic region of the stress–strain curve (stress plateau). At such strains, the levelling-induced stress change was normalized by the linearly increasing prestrain, and this results in the lowering of the calculated parameter.

**Figure 12:**
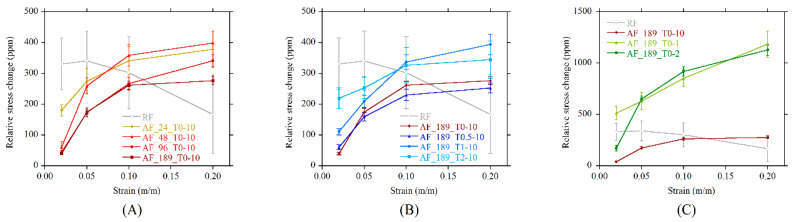
Relative stress change (RSC) under magnetic field versus prestrain as function of (**A**) magnetic field strength, (**B**) switch-on time, (**C**) switch-off time.

A further parameter was calculated, namely the relative stress change (RSC). It is a nondimensional parameter that represents the MF sensitivity of a smart foam with respect to the foam stiffness. It was calculated as the ratio between the MF-induced stress change and the compressive modulus of the foam. The RSC is higher in the RF compared to the AF systems at low prestrains due to the very low stiffness of the system (Figure 12A), but the huge variance and the superposition of RSC values at different prestrains rendered the RF as not suitable for sensing purposes. In AF systems, the sensitivity to MF increased with the prestrain level, and the reduced variance allowed for precise measurements. Smart foams produced under a low-strength MF have a higher RSC with respect to stiffer systems. A similar trend was detected in systems produced with delayed MF switch-on times (sON > 0). An increase in RSC with sON is clearly visible in Figure 12B, where the best performing system is the one characterized by thin and long aggregates (sON = 2). A very interesting behavior is shown by systems produced by switching off the magnetic field early (Figure 12C). RSC values for sOFF = 1 and sOFF = 2 are up to 3 times higher than RSC values calculated for sOFF = 10. Such samples are characterized by short and thick aggregates, and this result proves that this spatial configuration of the aggregates is the most effective for imparting a magnetosensitive behavior to smart foams.

Differently from conventional high density magnetoelastic materials, whose use is limited to less than a 5% strain, the smart foams show a monotonic growing trend up to a 20% strain. This opens up the possibility of using such materials in applications where a relevant sensitivity to MF is needed in a wide range of deformations.

## 4. Conclusions

It was confirmed that the use of the magnetic field during the foaming process of smart foams allowed us to field-structure the magnetic particles in linear aggregates along the magnetic field lines. Here, it was proven that the peculiar spatial distribution can be managed by properly changing the magnetic field intensity and its time profile. The smart foams exhibited a strong increase in the mechanical response along the magnetic field lines. Such result was dependent on the length and thickness of the developed aggregates.

Foams with aggregates of aligned particles exhibited magnetoelasticity (variation of the apparent elastic modulus, namely ΔE-effect) under a low intensity magnetic field. Such response was strain-dependent, continuous, and proportional to the intensity of the applied magnetic field, with a monotonic growing trend up to a 20% strain. Foams with longer and thicker aggregates exhibited the highest increase in stress change under magnetic field.

The compressive modulus variation under the MF (ΔE-effect) was more pronounced in systems with long and thick aggregates, but that trend showed a peak at the 5% prestrain and then decreased with the applied prestrain. Conversely, the relative sensitivity to MF (RSC), calculated as the stress change normalized by the sample stiffness, was higher in samples with short and thick aggregates, obtained by switching off the magnetic field early during the production process.

The balance between high magnetic field sensitivity and mechanical anisotropy was hence controlled by the shape features of the aggregates, which can be managed by properly changing the time profile of the magnetic field during the production process.

## Figures and Tables

**Table 1:** Summary of the investigated foaming process parameters in the samples series.

Sample Series	Investigated Parameter	Processing Conditions			
		CIP content (Volume %)	MF Strength (kA/m)	sON (min)	sOFF (min)
A	MF strength	3.3	0–275	0	10
B	sON	3.3	189	0–2	10
C	sOFF	3.3	189	0	0–2

**Table 2:** Compressive Young’s Modulus, yield stress, and yield strain of samples (reported as average ± error).

Series	Sample	Compressive Modulus (kPa)	Yield Stress (σ_y_) (kPa)	Strain at Yield (ε_y_) (m/m)
	PU	24.3 ± 1.5	1.99 ± 0.24	0.100
A	RF	30.1 ± 1.6	2.39 ± 0.37	0.100
	AF_24_T0-10	108.8 ± 2.8	7.95 ± 0.61	0.100
	AF_48_T0-10	146.8 ± 3.7	9.40 ± 0.73	0.100
	AF_96_T0-10	255.3 ± 3.7	13.65 ± 0.85	0.130 ± 0.011
	AF_189_T0-10	447.8 ± 7.6	14.85 ± 1.02	0.073 ± 0.008
B	AF_189_T0.5-10	302.2 ± 6.4	12.05 ± 0.72	0.105 ± 0.0007
	AF_189_T1-10	203.0 ± 2.3	10.50 ± 0.55	0.100
	AF_189_T2-10	76.9 ± 1.2	5.04 ± 0.38	0.100
C	AF_189_T0-1	41.0 ± 0.1	3.84 ± 0.4	0.100
	AF_189_T0-2	89.9 ± 0.3	8.22 ± 1.01	0.173 ± 0.016

**Figure A1:**
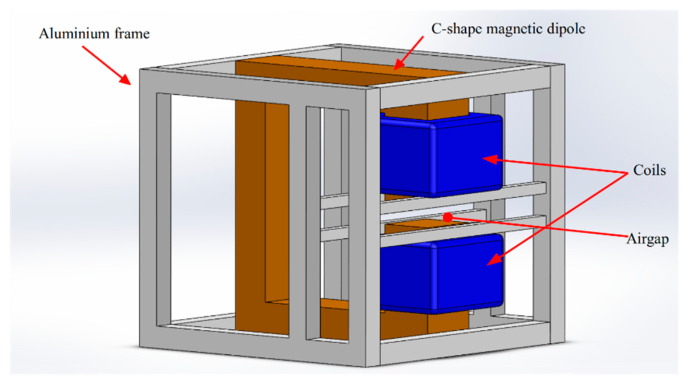
Electromagnet setup used for the production of foams with aligned particles.

**Figure A2:**
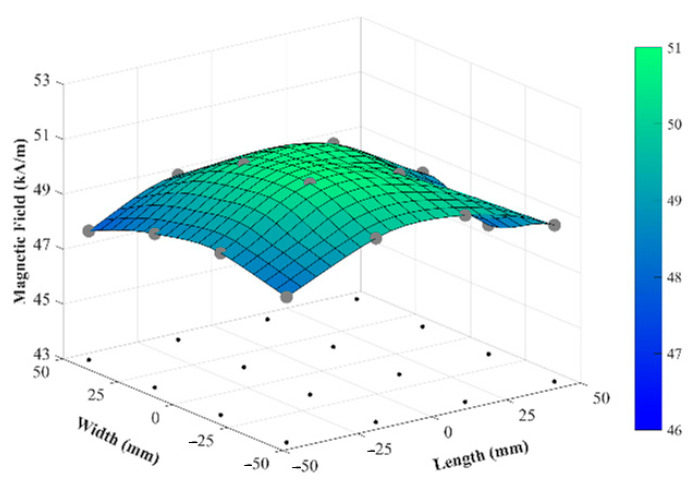
Magnetic field intensity evaluated in the electromagnet gap section. Grey points are the measured data; black points are the measurement position in the airgap; the color surface is the interpolated three-dimensional surface to guide the eye.

**Figure A3:**
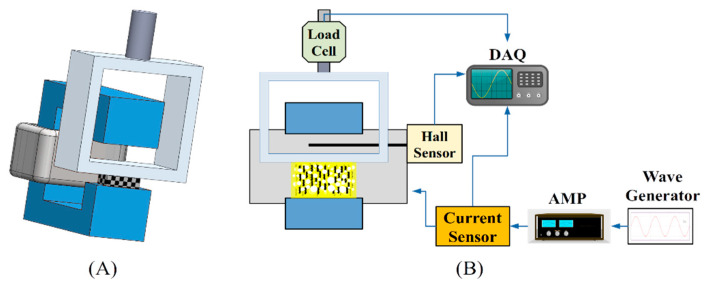
Experimental setup for the magnetoelastic characterization: (**A**) 3D sketch and (**B**) functional blocks.

## Appendix A

### Appendix A.1. Custom-Made Setup for the Production of Foams

The magnetic field time profile was generated by means of a custom made C-shape electromagnetic dipole, built from laminated iron sheets, with a 100 mm × 100 mm × 40 mm (length × width × height) airgap (Figure A1). Two custom made coils (1200 turns with a 1.2 mm diameter copper wire) were used to apply the magnetic induction field ranging between 0 and 275 kA/m. Coils were powered by a DC current power supply (model EA-PS 8065-10DT, EA-Elektro-Automatik GmbH & Co. KG, Viersen, Germany).

The magnetic field homogeneity in the airgap was preliminary checked in 16 points, evenly spaced in the electromagnet airgap section. A transverse Hall probe (model HMMT-6J04-VR, Lake Shore Cryotronics, Inc., Westerville, OH, USA) and a gaussmeter (model DSP 475, Lake Shore Cryotronics, Inc., Westerville, OH, USA) were used to measure the actual magnetic field. In Figure A2, we report the scattered measured data (grey data points) and an interpolated three-dimensional surface. The results show that the magnetic field intensity in the gap was almost homogeneous, with a variance lower than 3%.

### Appendix A.2. Custom-Made Setup for the Magnetoelastic Characterization

A specific setup was assembled to perform the magnetoelastic characterization of the smart foams (Figure A3). It consisted of an C-shaped electromagnetic dipole with an aperture of 30 mm and a poly(methyl methacrylate) (PMMA) frame for strain application and stress transfer to the load cell. A power amplifier BOP 100-4M (KEPCO, Inc., Flushing, NY, USA) and a waveform generator TGA12104 (Aim-Tti, Thurlby Thandar Instruments Limited, Huntingdon Cambridgeshire, UK) were used to generate the MF signal. The transverse Hall probe and the gaussmeter were also used to identify the actual strength of the applied MF. All signals (force, current, and magnetic field) were simultaneously recorded by means of an analog-to-digital data acquisition system (USB-6289, National Instruments Corporation, Austin, TX, USA). The PMMA frame was attached to a universal testing machine (LRX Plus, Lloyd Instruments AMETEK Inc., Berwyn, PA, USA) equipped with a 100 N load cell in order to measure the stress response of samples during the MF application.

### Appendix A.3. Macroscopic Features of Foams

Porosity was evaluated as the complement to 1 of the actual volume filled by solid phase and was calculated according to Equation (1):

(1) $$P=1-\frac{\rho_{f}}{\rho_{s}}$$

where *ρ_f_* and *ρ_s_* are the density of the foam sample and the density of the mixture before the foaming process, respectively. The evaluated density and calculated porosity are shown in Table A1.

**Table A1:** Composition, measured density, and porosity of samples.

Series	Sample	Particle Content (%)		Density (kg/m^3^)
		by Weight of Polymer	by Total Solid Volume	
	PU	0	0.0	64.4 ± 2.1
A	RF	25	3.3	88.0 ± 5.2
	AF_24_T0-10	25	3.3	91.5 ± 1.3
	AF_48_T0-10	25	3.3	87.8 ± 1.0
	AF_96_T0-10	25	3.3	92.0 ± 3.4
	AF_189_T0-10	25	3.3	91.6 ± 4.8
B	AF_189_T0.5-10	25	3.3	84.9 ± 2.7
	AF_189_T1-10	25	3.3	93.8 ± 1.9
	AF_189_T2-10	25	3.3	90.4 ± 2.2
C	AF_189_T0-1	25	3.3	87.1 ± 3.1
	AF_189_T0-2	25	3.3	81.6 ± 2.9
